# Can social support improve Mpox care-seeking among men who have sex with men through stigma reduction: a chain mediation analysis from China

**DOI:** 10.1186/s41256-025-00452-8

**Published:** 2025-10-01

**Authors:** Xin Ge, Yujie Liu, Shangbin Liu, Shunyu Tao, Chen Xu, Birong Wu, Ying Wang, Jiechen Zhang, Yong Cai

**Affiliations:** 1 Present Address: Public Health Research Center, Tongren Hospital, Shanghai Jiao Tong University School of Medicine, No.1111, Xianxia Road, Shanghai, 200336 China; 2 Center for Community Health Care, China Hospital Development Institute, Shanghai Jiao Tong University, No.227, South Chongqing Road, Shanghai, 200025 China; 3 Dermatology Department, Tongren Hospital, Shanghai Jiao Tong University School of Medicine, Shanghai, 200025 China

**Keywords:** Mpox, MSM, Healthcare-seeking intention, Stigma, Social support, Public health policy

## Abstract

**Background:**

Monkeypox (mpox) has emerged as a global public health concern, particularly among men who have sex with men (MSM). Stigma limits access to care, and the role of social support in shaping care-seeking through psychosocial mechanisms remains unclear. This study examined whether social support influences care-seeking intentions via stigma and perceived healthcare benefits among MSM in China.

**Methods:**

A cross-sectional study was conducted from October 2023 to March 2024 across 6 provinces in China. Descriptive statistics, chi-square tests, Spearman correlations, and logistic regression were performed to explore associations between HBM-related constructs and healthcare-seeking intentions. Structural equation modeling was used to examine the direct and indirect effects of social support via stigma and perceived healthcare benefits.

**Results:**

Among participants, 83.4% expressed an intention to seek healthcare for mpox. Directly, social support was positively associated with healthcare-seeking intention (β = 0.274, p < 0.001). Indirectly, social support affected healthcare-seeking intention through two pathways: (1) by reducing stigma (β = −0.108, p < 0.001), which in turn enhanced perceived healthcare benefits (β = −0.663, p < 0.001), ultimately increasing healthcare-seeking intention (chain effect β = 0.033, p = 0.005); and (2) directly enhancing perceived healthcare benefits (β = 0.091, p < 0.001), thereby increasing healthcare-seeking intention (β = 0.231, p < 0.001; indirect effect β = 0.042, p = 0.005). The total indirect effect accounted for 22.6% of the total effect.

**Conclusions:**

Social support enhances mpox care-seeking intention among MSM in China by reducing stigma and improving perceptions of privacy, affordability, and treatment efficacy. Integrated interventions—combining peer support, stigma reduction, and privacy protection—are needed to foster early health engagement.

## Introduction

Mpox, formerly known as monkeypox, is an emerging zoonotic disease caused by the Monkeypox virus (MPXV). Since 2022, the vast majority of mpox cases have been caused by clade IIb MPXV[1], with 99% occurring in adults outside Africa—89% among men who have sex with men (MSM) and 88% transmitted through sexual contact[2, 3]. In August 2024, the World Health Organization (WHO) re-declared mpox as a Public Health Emergency of International Concern (PHEIC), underscoring the urgent need for accessible, non-discriminatory healthcare services tailored to high-risk and socially marginalized populations.

As of March 2025, China had reported 2,909 confirmed mpox cases[3]. Although the overall case count remains lower than that of high-prevalence countries, the Chinese government has recognized the importance of early detection, behavioral interventions, and health equity in its "Healthy China 2030" national strategy. This strategy aligns with Sustainable Development Goal (SDG) 3.3, which aims to end the epidemics of HIV/AIDS and other communicable diseases by 2030[4]. Mpox, with its disproportionate impact on MSM populations and similarities in stigma-related care barriers, poses a significant challenge to achieving this goal, particularly in settings where social exclusion and health disparities remain entrenched.

Stigma in the context of sexually transmitted infections (STIs) is not a fixed individual attribute but a set of interactionally produced processes, including enacted degradation, discreditation and discrimination, whose meanings are constituted in concrete social encounters [5, 6]. In such settings, risk narratives centered on sexual behavior readily generate stigma by “othering” affected groups and assigning responsibility or perceived controllability to individuals [7, 8]. Among gay, bisexual, and other MSM, these dynamics intersect with pre-existing sexual-minority stigma to produce dual stigma [9, 10]. Furthermore, studies related to mpox have demonstrated that self-stigma (e.g., shame and guilt) and social stigma (e.g., social exclusion and discriminatory treatment) function as barriers to the utilization of healthcare services [7, 11–13].

Social support, defined as perceived emotional, informational, or instrumental assistance from others, serves as an important upstream determinant of health behavior in stigmatized contexts [14]. Robust support networks can alleviate the psychological burden associated with stigma, encourage adaptive coping strategies, and promote health-protective behaviors [15, 16]. However, among MSM [17], who frequently experience limited family and peer support as well as institutional mistrust, low levels of social support combined with high levels of stigma may exert a compounded negative effect. Inadequate supportive environments can intensify both self-stigma and social stigma, while perceptions of stigma may lead to concealment of illness and avoidance of healthcare services [18–20]. These dynamics ultimately reduce access to essential support networks and further exacerbate health disparities in this population.

**Fig. 1:**
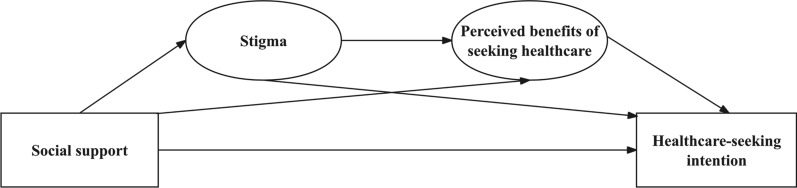
Hypothesized chain mediation model linking social support, stigma, perceived healthcare benefits, and healthcare-seeking intention

Based on the Health Belief Model, individuals’ health behaviors are shaped by their appraisal of the perceived benefits of taking action [21]. Evidence shows that among people living with STIs, perceptions of the effectiveness, affordability, and confidentiality of healthcare services influence decisions to seek treatment, and stigma often erodes these perceived benefits [9, 19, 22, 23]. Among MSM, supportive environments, including community networks, function as important channels for disseminating mpox health promotion materials and can improve knowledge of specific treatment options and isolation policies [16]. Based on these insights, we propose a chain-mediated model in which social support reduces stigmatization, thereby increasing the perceived benefits of treatment-seeking and, ultimately, the willingness to seek treatment (Fig. 1).

This study tests a chain-mediation model linking social support, stigma, and perceived healthcare benefits to care-seeking intentions among MSM in China—an under-researched population experiencing dual stigma and health disparities. By elucidating these psychosocial pathways, we aim to inform destigmatizing communication and privacy- and affordability-enhancing service strategies for mpox within China’s public health system, with implications for stigma-sensitive responses to emerging infectious diseases globally.

## Methods

### Study design

A nationwide cross-sectional study was conducted from October 2023 to March 2024 in 6 geographically representative provincial regions of China: Shanghai, Guangdong, Xinjiang, Shaanxi, Yunnan, and Liaoning. Participants were recruited using convenience sampling via local non-governmental organizations (NGOs) serving MSM communities. Anonymous questionnaires were completed through the online survey platform wenjuanxing (https://www.wjx.cn/).

### Study population

The inclusion criteria for participants were as follows: (1) male at birth; (2) aged 18 years or older; (3) residing in one of the selected six sites (having lived predominantly in the area for the past 6 months); (4) having sex with men in the past 6 months. The exclusion criteria are as follows: (1) no mpox diagnosis; (2) completion of the questionnaire in < 300 s; (3) incorrect answers to quality control questions; (4) IP address indicating a location outside the surveyed provincial regions.

### Variables and measurement

***Healthcare-seeking intentions for mpox*** were measured with a single item: “If you suspect you might have mpox, how willing are you to actively seek medical care?” Responses were rated on a 5-point scale ranging from “Very unwilling” to “Very willing”. “Willing” and “Very willing” were coded as “1”, while all other responses were coded as “0”.

***Social support*** was measured using the Perceived Social Support Scale (PSSS). Each item was rated on a 7-point Likert scale (1 = “Strongly disagree” to 7 = “Strongly agree”), with higher scores indicating stronger perceived support. The scale captures three sources of support: family, friends, and significant others (e.g., teachers, classmates, relatives). Internal consistency was excellent (Cronbach’s α = 0.947).

***Self-stigma*** was measured using 4 items adapted from the Internalized AIDS-related Stigma Scale [24, 25]: “I would feel guilty if I had mpox,”“I would feel ashamed,” “I would feel depressed,” and “I would feel worthless” (Cronbach's α = 0.886).

***Social stigma*** was assessed using 4 items reflecting societal norms [26]: “Most people think that people with mpox are unclean,” “should be ostracized,” “are disgusting,” and “lead promiscuous lives” (Cronbach's α = 0.947).

Self-stigma and social stigma were measured on a 1–5 Likert scale (with 1 = “Very low” and 5 = “Very high”).

***Perceived Healthcare Benefits*** are measured in terms of treatment efficacy, economic affordability, and privacy protection. Treatment efficacy was measured with 5 items assessing confidence in mpox treatment, perceived competence of healthcare providers, the effectiveness of medication, appropriateness of treatment methods, and the likelihood of positive health outcomes. The economic affordability was assessed with 4 items covering anticipated treatment expenses, potential work disruption, income loss, and perceived financial burden on the family. Privacy protection was evaluated with 5 items addressing concerns about confidentiality during healthcare visits, potential exposure of personal health information, risk of being identified by others, disclosure of sexual orientation, and perceived social stigma. These variables were measured on a 5-point Likert scale (1 = Strongly Disagree, 5 = Strongly Agree). As all items were negatively worded, scores were reverse-coded (i.e., 1 = 5, 2 = 4, etc.), such that higher scores indicate greater perceived benefits (Cronbach's α = 0.949).

***Participant Characteristics.*** The variables included age, education level (lower secondary or below, upper secondary, tertiary and above; equivalent to junior high school and below [typically completed at age 15], senior high school [completed at age 18], and college/university or above in the Chinese education system), marital status (single, married, divorced/widowed), monthly income (CNY ≤ 3000, 3001–6000, 6001–12000, ≥ 12,001), employment status (mental labor, manual labor, student, freelance work, unemployed), length of residence in the surveyed area (local resident, < 5 years, > 5 years), sexual orientation (homosexual and others). HIV status was assessed as a binary variable (0 = no, 1 = yes). Sexual risk behaviors were classified as low-risk (≤ 1 partner AND consistent condom use in the past 6 months), moderate-risk (meeting one criterion), or high-risk (meeting neither criterion).

### Statistical analysis

Descriptive analyses were performed to examine sociodemographic and psychosocial characteristics (N = 2,347). Socioeconomic status (SES) was categorized using latent class analysis (LCA) based on three indicators: educational attainment, occupation type, and monthly income. Competing models with two to four latent classes were tested and compared using Akaike Information Criterion (AIC), Bayesian Information Criterion (BIC), and model deviance (G^2^). The three-class model was selected as optimal, yielding the lowest BIC (14,272.22) and a substantial reduction in deviance (from 439.28 in the two-class model to 74.33). The resulting classes were labeled as “Low SES,” “Middle SES,” and “High SES,” and were subsequently included as a categorical covariate in the structural equation model. Detailed LCA results are presented in Appendix A.

For descriptive analyses, continuous psychosocial variables—including treatment efficacy, economic affordability, privacy protection, self-stigma, and social stigma—were dichotomized into “low” and “high” categories using the sample median as the cutoff point. Chi-square tests were conducted to examine associations between healthcare-seeking intention and categorical sociodemographic and psychosocial variables. Additionally, Spearman correlation analyses were used to assess the relationships among key psychosocial variables, as well as their correlations with mpox healthcare-seeking intention. All descriptive analyses and LCA were conducted using R software (version 4.2.1).

In the mediation model, self-stigma and social stigma were combined to construct the latent variable of stigma, while treatment concerns, economic concerns, and exposure concerns were combined into the latent variable of perceived healthcare benefits. Measurement validity of stigma and perceived healthcare benefits was tested using confirmatory factor analysis (CFA). The model was tested using structural equation modeling in Mplus (version 8.0). All models were estimated using the maximum likelihood estimator with robust standard errors (MLR) to account for non-normality. STDYX-standardized coefficients were reported. A two-sided p-value < 0.05 was considered statistically significant.

## Results

### Characteristics of the participants

In this study, a total of 2,481 questionnaires were collected, of which 2,403 were valid (effective rate: 96.86%), and 2,347 participants were included in the study. Among them, 23.3% were from Shanghai, 20.9% from Guangzhou, 21.0% from Shenyang, 8.2% from Xi'an, 13.1% from Kunming, and 13.5% from Urumqi.

Table 1 presents the descriptive statistics of the 2,347 participants, of whom 1,970 (83.4%) expressed willingness to seek healthcare for mpox. The majority were young adults aged 18–34 (74.2%), were single (85.1%), identified as homosexual (78.7%), and reported low SES (62.1%). Most were HIV-negative (92.2%) and engaged in 1–2 high-risk sexual behaviors (67.1%). Over half exhibited high levels of self-stigma (50.9%) and social stigma (53.8%), while 43.0% reported high social support. A total of 71.4%, 32.8%, and 44.6% of participants reported high levels of perceived treatment efficacy, economic affordability, and privacy protection, respectively. All variables, except for employment, length of residence, and HIV status, were significantly associated with healthcare-seeking intention for mpox (p < 0.05).

**Table 1:** Sociodemographic and Psychosocial Characteristics by Mpox Healthcare-Seeking Intention (N = 2,347)

Variables	Overall (N = 2,347)	Help-seeking intention for mpox		*χ*^*2*^	p
		Yes (N = 1,970)	No (N = 377)		
**Age**				15.746	0.001^**^
18–24	541 (23.1)	441 (22.4)	100 (26.5)		
25–34	1200 (51.1)	1038 (52.7)	162 (43.0)		
35–44	456 (19.4)	377 (19.1)	79 (21.0)		
≥ 45	150 (6.4)	114 (5.8)	36 (9.5)		
**Marital status**				19.225	< 0.001^***^
Single	1997 (85.1)	1702 (86.4)	295 (78.2)		
Married	238 (10.1)	177 (9.0)	61 (16.2)		
Divorced/widowed	112 (4.8)	91 (4.6)	21 (5.6)		
**SES**					
Low	470 (20.0)	368 (18.7)	102 (27.1)	20.929	< 0.001^***^
Middle	1458 (62.1)	1262 (64.1)	196 (52.0)		
High	419 (17.9)	340 (17.3)	79 (21.0)		
**Length of residence**					
Local	912 (38.9)	748 (38.0)	164 (43.5)	4.076	0.130
< 5 year	848 (36.1)	722 (36.6)	126 (33.4)		
≥ 5 years	587 (25.0)	500 (25.4)	87 (23.1)		
**Sexual orientation**					
Homosexual	1846 (78.7)	1570 (79.7)	276 (73.2)	7.928	0.005^**^
Others	501 (21.3)	400 (20.3)	101 (26.8)		
**HIV status**				0.571	0.450
No	2164 (92.2)	1820 (92.4)	344 (91.2)		
Yes	183 (7.8)	150 (7.6)	33 (8.8)		
**High-risk sexual behavior**				11.305	0.004^**^
Low	772 (32.9)	674 (34.2)	98 (26.0)		
Middle	1021 (43.5)	849 (43.1)	172 (45.6)		
High	554 (23.6)	447 (22.7)	107 (28.4)		
**Treatment efficacy**				39.033	< 0.001^***^
Low	671 (28.6)	513 (26.0)	158 (41.9)		
High	1676 (71.4)	1457 (74.0)	219 (58.1)		
**Economic affordability**				17.099	< 0.001^***^
Low	1578 (67.2)	1290 (65.5)	288 (76.4)		
High	769 (32.8)	680 (34.5)	89 (23.6)		
**Privacy protection**				27.274	< 0.001^***^
Low	1300 (55.4)	1045 (53.0)	255 (67.6)		
High	1047 (44.6)	925 (47.0)	122 (32.4)		
**Self Stigma**				9.360	0.002^**^
Low	1153 (49.1)	995 (50.5)	158 (41.9)		
High	1194 (50.9)	975 (49.5)	219 (58.1)		
**Social stigma**				14.943	< 0.001^***^
Low	1085 (46.2)	945 (48.0)	140 (37.1)		
High	1262 (53.8)	1025 (52.0)	237 (62.9)		
**Social Support**				99.798	< 0.001^***^
Low	186 (7.9)	124 (6.3)	62 (16.4)		
Middle	1151 (49.0)	920 (46.7)	231 (61.3)		
High	1010 (43.0)	926 (47.0)	84 (22.3)		

^*^P < 0.05, **P < 0.01, ***P < 0.001

### Spearman Correlations Among Psychosocial Variables and Mpox Healthcare-Seeking Intention

Table 2 shows that help-seeking intention for mpox was significantly correlated with all measured variables (p < 0.01). Social support was significantly negatively correlated with self-stigma (r = -0.068, p < 0.01) and social stigma (r = -0.044, p < 0.05). Moreover, social support was significantly and positively associated with key components of perceived benefits, including treatment efficacy (r = 0.091, p < 0.01), economic affordability (r = 0.145, p < 0.01), and privacy protection (r = 0.118, p < 0.01). A significant positive correlation was also found between social support and help-seeking intention for mpox (r = 0.240, p < 0.01).

**Table 2:** Spearman Correlations Among Psychosocial Variables and Mpox Healthcare-Seeking Intention

Variables	Self-stigma	Social stigma	Treatment efficacy	Economic affordability	Privacy protection	Social support	Help-seeking intention for mpox
Self-stigma	1						
Social stigma	0.539^**^	1					
Treatment efficacy	−0.313^**^	−0.315^**^	1				
Economic affordability	−0.402^**^	−0.389^**^	0.610^**^	1			
Privacy protection	−0.497^**^	−0.511^**^	0.575^**^	0.801^**^	1		
Social support	−0.068^**^	−0.044^*^	0.091^**^	0.145^**^	0.118^**^	1	
Help-seeking intention for mpox	−0.082^**^	−0.105^**^	0.165^**^	0.156^**^	0.164^**^	0.240^**^	1

^*^P < 0.05, **P < 0.01

### Path Analysis

To assess construct validity, a confirmatory factor analysis (CFA) was conducted on the latent variables Stigma and Perceived Healthcare Benefits. The model exhibited acceptable fit: χ^2^ (4) = 146.064, p < 0.001; CFI = 0.976; TLI = 0.940; SRMR = 0.021. RMSEA was higher than ideal (0.123, 90% CI [0.106, 0.141]), likely due to the model’s low degrees of freedom. Factor loadings ranged from 0.688 to 0.934, and all subscales demonstrated high internal consistency (Cronbach’s α > 0.88). CFA details are provided in Appendix B.

**Fig. 2:**
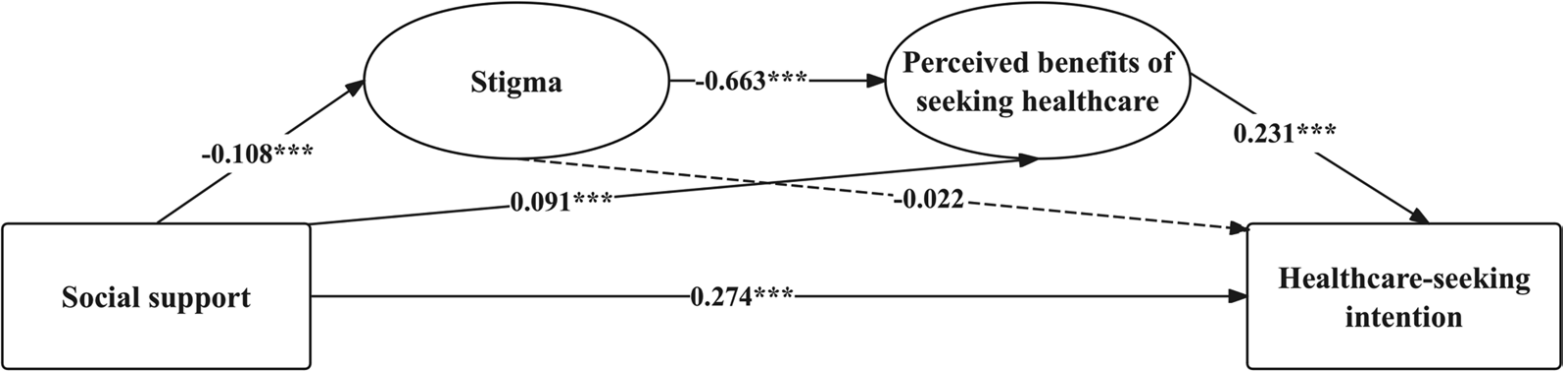
Chain mediation model of social support and healthcare-seeking intention. Adjusted for age, SES, marital status, sexual orientation, HIV status, and high-risk sexual behavior. Standardized path coefficients are shown. ***p < 0 .001

The results of the chain mediation model, as shown in Table 3, 4 and Fig. 2, indicate that social support has both direct and indirect effects on healthcare-seeking intention for mpox. The direct effect of social support on healthcare-seeking intention was significant (β = 0.274, p < 0.001). Additionally, social support exerted an indirect effect through two mediated pathways: (1) via stigma (β = -0.018, p < 0.001) and perceived healthcare benefits (β = -0.663, p < 0.001), which subsequently increased healthcare-seeking intention (β = 0.231, p < 0.001), resulting in a significant chain indirect effect (β = 0.033, p = 0.005); and (2) via perceived healthcare benefits alone (β = 0.091, p < 0.001), also leading to higher intention( β = 0.231, p < 0.001). The total indirect effect was significant (β = 0.080, p < 0.001), accounting for approximately 22.6% of the total effect. Stigma alone did not directly affect healthcare-seeking intention (β = -0.022, p = 0.706), but played a meaningful role within the sequential mediation chain.

**Table 3:** Standardized path coefficients of the chain mediation model

Path	β	S.E	95%CI	p
X → M1	− 0.108	0.028	[− 0.163, − 0.053]	< 0.001^***^
M1 → M2	− 0.663	0.025	[− 0.712, − 0.614]	< 0.001^***^
M2 → Y	0.231	0.057	[0.121, 0.344]	< 0.001^***^
X → M2	0.091	0.022	[0.048, 0.134]	< 0.001^***^
M1 → Y	− 0.022	0.057	[− 0.141, 0.083]	0.706
X → Y	0.274	0.029	[0.208, 0.322]	< 0.001^***^

^***^P < 0.001

Adjusted for age, SES, marital status, sexual orientation, HIV status, and high-risk sexual behavior

X = Social Support, M1 = Stigma, M2 = Perceived benefits of seeking healthcare, Y = Healthcare-Seeking Intention

**Table 4:** Total, direct, and indirect effects in the chain mediation model

Effect type	Path	*β*	S.E	**95% CI**	p
Total effect	X → Y (total)	0.354	–	–	–
Direct effect	X → Y	0.274	0.029	[0.208, 0.322]	< 0.001^***^
Total indirect effect	Sum of all indirect effects	0.080	0.007	[0.043, 0.117]	< 0.001^***^
Indirect effect (path 1)	X → M1 → M2 → Y	0.033	0.008	[0.009, 0.057]	0.005^**^
Indirect effect (path 2)	X → M1 → Y	0.005	0.012	[-0.002, 0.029]	0.708
Indirect effect (path 3)	X → M2 → Y	0.042	0.015	[0.013, 0.071]	0.005^**^

^**^P < 0.01, ***P < 0.001

Adjusted for age, SES, marital status, sexual orientation, HIV status, and high-risk sexual behavior

X = Social Support, M1 = Stigma, M2 = Perceived benefits of seeking healthcare, Y = Healthcare-Seeking Intention

## Discussion

This study identified stigma as a key psychological bridge linking social support to mpox healthcare-seeking intention among MSM in China. Through a chain mediation model, we found that social support reduces both self-stigma and social stigma, enhancing individuals' perceived benefits of seeking healthcare, ultimately increasing their willingness to engage with medical services. Notably, stigma did not exert a direct suppressive effect on intention but rather functioned as an attitudinal filter that influences how individuals cognitively evaluate the value of care. These findings provide empirical evidence supporting stigma as the “bridge” variable, mediating the influence of upstream social support on downstream behavioral intention.

The perceived healthcare benefits emerged as crucial determinants of healthcare-seeking intentions, particularly the dimension of privacy protection. Although treatment efficacy and affordability also played significant roles, especially among lower socioeconomic groups, the fear of identity exposure and public judgment was the most salient barrier. These findings echo earlier studies on stigmatized conditions such as HIV, in which anticipated discrimination often outweighs clinical or economic considerations when individuals decide whether to seek care [27, 28]. Privacy, therefore, appears to be a threshold determinant for care-seeking behavior in stigmatized populations.

Contrary to earlier research indicating stigma’s direct suppressive effect on healthcare-seeking behaviour [29, 30], our study did not identify such a direct association. This discrepancy may be attributed to specific contextual factors surrounding mpox, such as its perceived manageability compared to HIV and the post-COVID-19 emphasis on anonymous care [16, 31]. The framing of mpox as a “homosexual disease” in both media and public discourse has likely intensified stigma, yet redirected its effect through distorted risk–benefit evaluations rather than outright avoidance [32]. Similar patterns were documented in the U.S. during the 2022–2023 mpox outbreak, where stigma shaped perceived costs and benefits of care more than behavior itself [33].

The role of social support is pivotal, as evidenced by HIV-related research highlighting supportive peer networks' effectiveness in enhancing health resource utilization and mitigating stigma [34]. Similarly, social networks have been shown to be effective in low-incidence settings like China, facilitating early identification of exposure and reducing associated anxiety and stigma [35]. Strengthening community connectedness, as demonstrated during the US mpox outbreak, significantly improved access to reliable health information and reduced health anxiety among sexual minorities [36–38]. Strengthening community connectedness not only buffers stress but also promotes health-protective behaviors by enhancing informational access and emotional resilience. Additionally, individuals infected with mpox often experience complex psychosocial challenges during illness and isolation, highlighting the value of combining high-quality clinical care with psychosocial support as an integrated approach to mpox response [39].

Consistent with our survey finding of a high willingness to seek care (83.4%), prior studies have likewise observed that most MSM in China intend to seek medical attention [40]. Nevertheless, delays in presentation are common. Moreover, only 52.5% of MSM reported willingness to fully comply with isolation requirements, which is suboptimal for effective outbreak control. Such results may be related to the fact that mpox has not yet attracted sufficient public attention in China, and knowledge of its severity and treatment measures is still limited [41, 42]. The importance of outreach and public education focusing on mpox is emphasized to increase awareness of mpox, to strengthen the understanding of the benefits of timely treatment, and to promote earlier participation in health care services.

These findings have important public health implications. First, enhancing social support networks, particularly those that affirm identity and reduce isolation, may indirectly reduce stigma and improve care uptake. Specifically in China, this can be operationalized through a dual-layered support network combining peer-led health advocates (e.g., training MSM community leaders to disseminate care-seeking experiences) and institutionalized medical navigators to guide anonymous service access—a model that could be embedded into the national "Integration of Prevention and Treatment" pilot program. Second, efforts to combat stigma should go beyond awareness campaigns and focus on reshaping structural and interpersonal norms that reinforce discrimination. Two context-driven strategies are critical: (1) updating existing healthcare data protection regulations to prohibit unauthorized disclosure of sensitive personal information, and (2) scaling up community-anchored anonymous clinics that decouple identity from service delivery through technology-enhanced anonymous registration systems. Third, policy interventions must prioritize privacy safeguards while integrating psychosocial support with clinical care. Lessons from the UK underscore the effectiveness of coordinated collaboration between community-based organizations and sexual health clinics, which could be adapted to China’s context through public-hospital-NGO partnerships [38]. Globally, three actionable pathways emerge: (1) Reframing health communication to emphasize risk behaviors (e.g., multi-partner contact) rather than identity labels, aligning with WHO’s 2022 mpox guidance to reduce media-driven stigmatization [43]; (2) Establishing a WHO-led anti-stigma accreditation system for healthcare providers, mandating cultural competence training on socially marginalized groups health needs as part of continuing medical education [44]; and (3) Deploying a transnational stigma surveillance platform (e.g., Global Stigma Index) to dynamically link social support levels with outbreak responses, enabling targeted resource allocation—for instance, directing mobile clinics to high-stigma regions or AI-driven mental health chatbots to low-support areas, drawing on the regional mpox surveillance network established in Central Africa [45].

Despite sampling from six geographical regions in China with a relatively large sample size, this study has several limitations. Its cross-sectional design precludes causal inference, and reliance on self-reported data introduces the possibility of social desirability and recall bias, especially in the context of stigma or risk behavior reporting. Though geographically diverse, the sample may not fully represent subpopulations such as older MSM or those without internet access. Additionally, as participants were recruited through MSM-serving NGOs, selection bias may have occurred—those engaged with such organizations may have greater access to health information and social support, potentially inflating care-seeking intentions compared to more marginalized individuals. While our model controlled for key sociodemographic variables, unmeasured confounders may still exist. Furthermore, healthcare-seeking intention was assessed using a single-item indicator, which may not fully capture the multidimensional nature of behavioral intentions or actual healthcare utilization.

Future studies should adopt longitudinal designs to validate the proposed causal pathways and examine the temporal dynamics between social support, stigma, and perceived healthcare benefits. Experimental and intervention-based studies are also needed to assess the real-world effectiveness of stigma-reduction strategies, privacy-enhancing measures, and support-building interventions in improving timely care-seeking behavior. In addition, sampling frames should extend beyond formal organizations—using venue-based, respondent-driven, and platform-based outreach—to capture a broader spectrum of social-support contexts and improve generalizability. Moreover, evaluating these mechanisms across diverse cultural and healthcare settings can help generate context-sensitive, scalable models for stigma-sensitive health promotion, particularly for vulnerable populations facing emerging infectious disease threats.

## Conclusions

This study highlights the indirect yet significant role of social support in promoting mpox care-seeking among MSM through stigma reduction and improved perception of healthcare benefits. Stigma indirectly shaped care-seeking intentions by influencing evaluations of healthcare benefits, with privacy protection emerging as a key factor. These findings highlight the need for stigma-sensitive interventions that integrate peer support, privacy safeguards, and culturally competent care. Policy efforts should prioritize anonymous services and community engagement to address the unique needs of stigmatized populations in China and beyond.

## Data Availability

The datasets generated and/or analyzed during the current study are not publicly available due to concerns related to participant confidentiality and the sensitive nature of the data involving stigmatized populations, but are available from the corresponding author on reasonable request.

## Appendix A

See Tables

**Table 5:** Latent Class Analysis for Socioeconomic Status (SES)

Number of classes	AIC	BIC	G^2^ (deviance)
2 Classes	14,469.39	14,567.32	439.28
3 Classes	14,122.43	14,272.22	**74.33**
4 Classes	14,091.88	14,293.51	25.77

^5^,

**Table 6:** Conditional Probabilities of SES Indicators by Latent Class

Indicator	Most probable category	Low SES (Class 1)	Middle SES (class 2)	High SES (class 3)
Education	Low (≤ junior high)	95.3%	51.7%	92.2%
Income	Low (≤ 3000 RMB)	49.7%	56.0%	81.6%
Occupation	Manual labor	38.0%	50.9%	71.8%

6

## Appendix B

See Table

**Table 7:** Confirmatory Factor Loadings of Latent Constructs

Latent Variable	Indicator	Standardized Loading	SE	p-value
Stigma	Self-stigma	0.721	0.021	< 0.001
	Social stigma	0.726	0.020	< 0.001
Perceived Benefits	Treatment efficacy	0.688	0.016	< 0.001
	Economic affordability	0.903	0.009	< 0.001
	Privacy protection	0.934	0.008	< 0.001

Model fit: χ^2^(4) = 146.064, p < .001; CFI = 0.976; TLI = 0.940; RMSEA = 0.123 (90% CI = [0.106, 0.141]); SRMR = 0.021

^7^

## Abbreviations

MPXV: Monkeypox virus
(WHO): World Health Organization
(PHEIC): Public Health Emergency of International Concern
(MSM): Men who have sex with men
(STIs): Sexually transmitted infections
(NGOs): Non-governmental organizations
(PSSS): The Perceived Social Support Scale
(SES): Socioeconomic status
(LCA): Latent class analysis
(AIC): Akaike Information Criterion
(BIC): Bayesian Information Criterion
(CFA): Confirmatory factor analysis
(MLR): The maximum likelihood estimator with robust standard errors
